# Thermodynamic data of phenol adsorption on chemically modified activated carbons

**DOI:** 10.1016/j.dib.2020.105240

**Published:** 2020-02-04

**Authors:** Liliana Giraldo, Valentina Bernal Fernandez, Juan Carlos Moreno-Piraján

**Affiliations:** a Departamento de Química, Universidad Nacional de Colombia, Bogotá, Colombia; b Departamento de Química, Universidad de los Andes, Bogotá, Colombia

**Keywords:** Adsorption, Entropy, Gibbs energy, Immersion enthalpy, Interaction enthalpy, Phenol

## Abstract

The presence of phenol in water bodies exists due to the discharge of wastewater from industrial, agricultural and domestic activities. Its presence in water is associated with a decrease in the quality of drinking water because its change the taste and odour [1]. The adsorption process is one of the most used treatments to remove the phenol of waters and the activated carbon is an appropriate adsorbent due to its high surface area, porosity and low cost.

The studies about the adsorption process are addressed by different views of point such as equilibrium and thermodynamic data. In this work, the adsorption isotherms of phenol on five activated carbons with different physicochemical properties in aqueous solution are presented. In addition, the immersion enthalpies, the interaction enthalpies, the Gibbs energy and the entropy changes are included.

The isotherms data are adjusted to the Freundlich and Sips models. The immersion enthalpy values are between −7.670 and −57.0 J g^−1^, the interaction enthalpies are *between* 48.00 and −11.70 J g^−1^, the Gibbs energy change are between −5337 and −12322 J mol^−1^ K^−1^ and finally, the entropy change values are between 18.10 and 39.70 J K^−1^.

**Table:** Specifications Table

Subject area	*chemistry*
More specific subject area	*Adsorption and thermodynamic.*
Type of data	*Table, figure*
How data was acquired	*The adsorption capacity was determined by matter balance. The initial and final concentrations of phenol are determined with UV-VIS spectrophotometry.* *The immersion calorimetries are determined with immersion calorimetry.* *The interaction enthalpies, Gibbs energies and entropies are determined by use of Hess Law, equilibrium constant and Gibbs-Helmholtz equation, respectively.*
Data format	*analyzed*
Experimental factors	*A commercial activated carbon (AC) was thermally treated at 1073,1173 and 1273 K in a Thermolyne furnance with N*_*2*_*atmosphere. An oxidation with Nitric acid 6 M was made in the AC.* *The adsorption isotherms are determined in phenols solutions with concentrations between 0.11- 10.*6 mmol L^−1^*at 293 K.* *The immersion calorimetries are determined in a Tyan calorimeter at 293 K.*
Experimental features	*Phenol adsorption on activated carbon from aqueous solution.*
Data source location	*Universidad Nacional de Colombia, Bogotá, Colombia.*
Data accessibility	*Data are accessible with the article*
Related research article	*Not applied*

**Table:** 

**Value of the Data** • The physicochemical properties of activated carbons allow to determine the adsorbent potential of different organic compounds. • Adsorption of phenol on activated carbon has a good potential application in the alimentary industry. • The isotherm data are useful for predicting the adsorption capacity in activated carbons with different physicochemical properties. The adsorption mechanism can be studied by determination of the best fit isotherm model. • The thermodynamic data complement the adsorption isotherms and can corroborate the adsorption mechanism.

## 1 Data

The physicochemical properties of activated carbon can be found in Table 1.

**Table 1:** Physicochemical characteristics of the activated carbons AcOx, Ac, AC1073, AC1173 and AC1173.

Activated carbon	Surface Area (m^2^ g^−1^)	Microporous volume (cm^3^g^−1^)	Total acidity (μmol g^−1^)	Total basicity (μmol g^−1^)	pH_pzc_
ACox	469	0.18	656 ± 32.8	735 ± 36.8	3.40
AC	864	0.35	90.5 ± 4.53	742 ± 37.1	5.40
AC1073	1127	0.48	93.6 ± 4.68	1210 ± 60.5	11.1
AC1173	814	0.34	93.0 ± 4.65	2037 ± 102	8.90
AC1273	711	0.30	94.1 ± 4.75	2290 ± 115	9.96

**Fig. 1:**
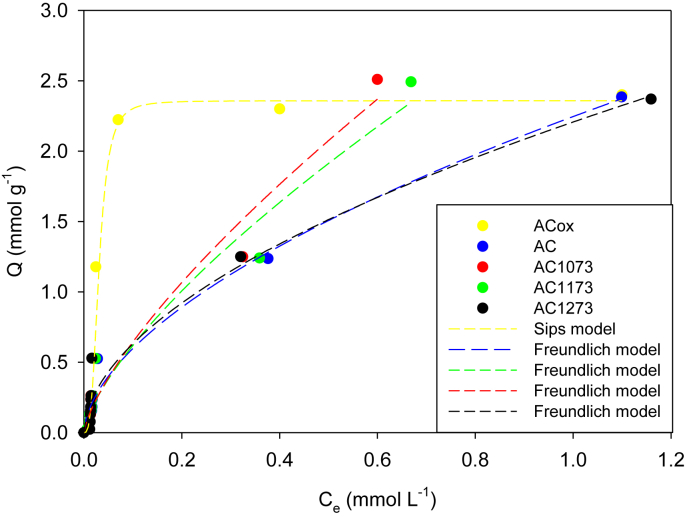
Adsorption of Phenol on activated carbons (AcOx, Ac, AC1073, AC1173 and AC1173) in aqueous solutions at 293 K. The data were adjusted to the Freundlich and Sips models.

**Fig. 2:**
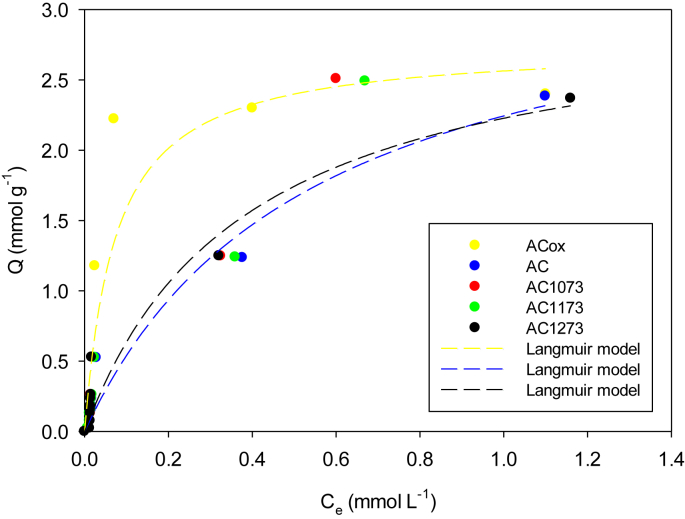
Adsorption of Phenol on activated carbons (AcOx, Ac, AC1073, AC1173 and AC1173) in aqueous solutions at 293 K. The data were adjusted to the Langmuir model.

The isotherms adsorption data of phenol in activated carbons AcOx, AC, AC1073, AC1173 and AC1173 are presented in Fig. 1. The adsorption data were adjusted at Langmuir (Fig. 2), Sips or Freundlich models according to the best R^2^, the mathematical parameters of the models are presented in Table 2, Table 3.

**Table 2:** Parameters of the Sips or Freundlich models applied to adsorption isotherms of phenol on the activated carbons AcOx, Ac, AC1073, AC1173 and AC1173.

Activated carbon	model	Q_m_	K	n	R^2^
ACox	SIPS	2.36	32.2	0.31	0.97
AC	Freundlich	–	2.25	0.58	0.98
AC1073	Freundlich	–	3.11	0.70	0.96
AC1173	Freundlich	–	3.44	0.73	0.96
AC1273	Freundlich	–	2.21	0.54	0.97

**Table 3:** Parameters of the Langmuir model applied to adsorption isotherms of phenol on activated the carbons AcOx, Ac, AC1073, AC1173 and AC1173.

Activated carbon	Q_m_ (mmol g^−1^)	K (mmol g^−1^)	R^2^
AcOx	2.30	6.88	0.95
AC	1.86	6.41	0.96
AC1073	NA	NA	NA
AC1173	NA	NA	NA
AC1273	2.60	8.03	0.95

The immersion enthalpies in water and solution of phenol were determined at 293 K, the results can be observed in Table 4, Table 5, respectively.

**Table 4:** Immersion enthalpies of the activated carbons AcOx, Ac, AC1073, AC1173 and AC1173 in water at 293 K.

Activated Carbon	ΔH_imm_ ACox (J g^−1^)	ΔH_imm_ AC (J g^−1^)	ΔH_imm_ AC1073 (J g^−1^)	ΔH_imm_ AC1173 (J g^−1^)	ΔH_imm_ AC1273 (J g^−1^)
H_2_O	−66.6	−49.7	−27.4	−32.4	−31.5

**Table 5:** Immersion enthalpies of the activated carbons AcOx, Ac, AC1073, AC1173 and AC1173 in phenol solutions with concentrations between 0.74 and 10.6 mmol L^−1^ at 293 K.

C_o_ Phenol (mmol L^−1^)	ΔH_imm_ ACox (J g^−1^)	ΔH_imm_ AC (J g^−1^)	ΔH_imm_ AC1073 (J g^−1^)	ΔH_imm_ AC1173 (J g^−1^)	ΔH_imm_ AC1273 (J g^−1^)
0.74	−24.7 ± 0.49	−7.65 ± 0.15	−11.6 ± 0.23	−13.9 ± 0.28	−31.1 ± 0.62
1.06	−19.3 ± 0.39	−7.71 ± 0.16	−12.9 ± 0.26	−8.13 ± 0.16	−29.2 ± 0.58
2.13	−41.1 ± 0.82	−24.3 ± 0.49	−33.4 ± 0.67	−35.0 ± 0.70	−19.2 ± 0.38
5.31	−33.6 ± 0.67	−39.4 ± 0.79	−35.4 ± 0.71	−27.6 ± 0.55	−24.9 ± 0.50
10.6	−57.0 ± 1.14	−39.2 ± 0.79	−39.3 ± 0.79	−44.4 ± 0.89	−25.9 ± 0.52

**Fig. 3:**
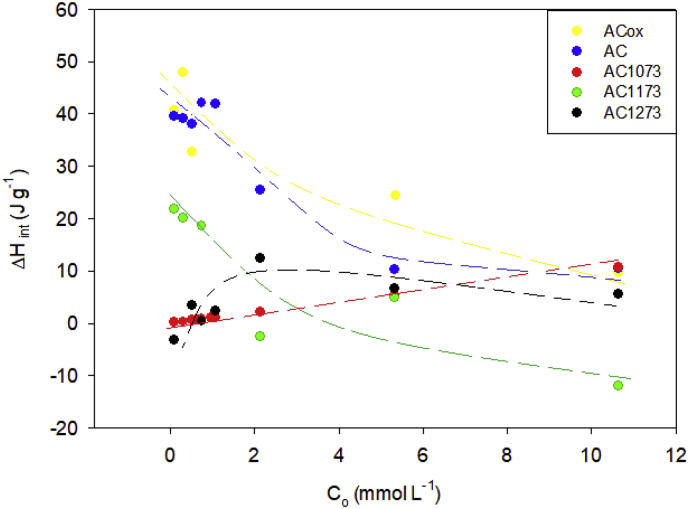
Interaction enthalpies of the activated carbons AcOx, Ac, AC1073, AC1173 and AC1173 in phenol solutions with concentrations between 0.74 and 10.6 mmol L^−1^ at 293 K.

**Fig. 4:**
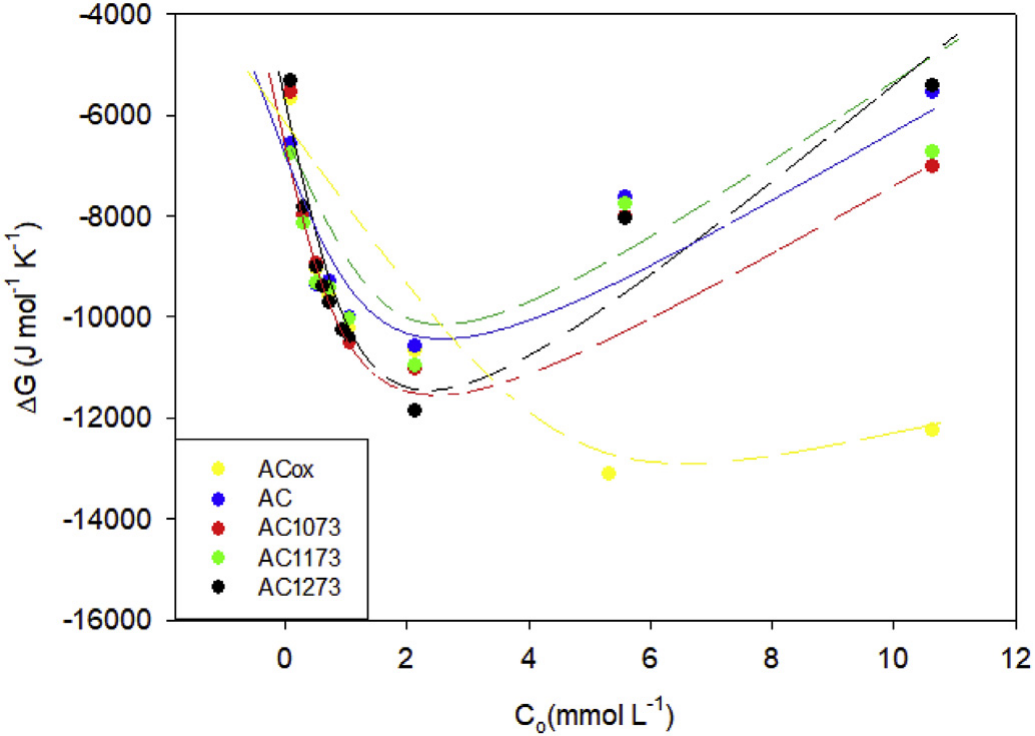
Gibbs energy change of the activated carbons AcOx, Ac, AC1073, AC1173 and AC1173 in phenol solutions with concentrations between 0.74 and 10.6 mmol L^−1^ at 293 K.

**Fig. 5:**
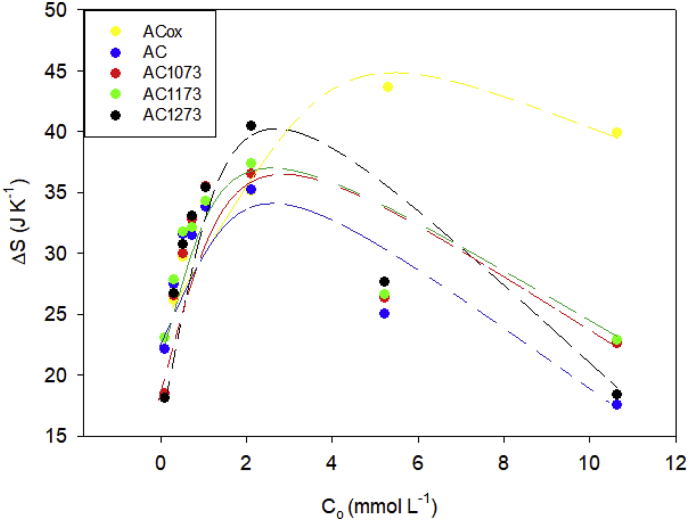
Entropy change of the activated carbons AcOx, Ac, AC1073, AC1173 and AC1173 in phenol solutions with concentrations between 0.74 and 10.6 mmol L^−1^ at 293 K.

The thermodynamic parameters such as the interaction enthalpy, Gibbs energy and entropy for the adsorption of phenol on activated carbons at concentration between 0.74 and 10.6 mmol L^−1^ are presented in Fig. 3, Fig. 4, Fig. 5 respectively.

## 2 Experimental design, materials, and methods

### 2.1 Activated carbons

Five activated carbons that differ in their physicochemical characteristics were used as adsorbents. Activated carbon AC is a commercial activated carbon brand CARBOCHEM BRAND GS50 (CARBOCHEM INC., Philadelphia, PA, USA) prepared from coconut shell and physical activation with CO_2_. It was conditioning for the use with a washed in diluted HCl solution and distilled water until constant pH, then dried at 373 K.

Activated carbon AC was subjected to a thermal treatment at 1073, 1173 K and 1273 K to modified the physicochemical characteristics of the initial activated carbon. The samples obtained were named as AC 1073, AC1173 and AC1273.

For the procedure, the activated carbon AC was put in a THERMOLYNE furnace. 100 g of AC activated carbon are deposited and left for 2 h at the assigned temperature with a 2 K s^−1^ heating ramp in a nitrogen atmosphere. Afterwards, the activated carbon is cooled in the furnace and stored in amber glass jars with airtight seal.

The ACox is an oxidized activated carbon and was produced by treatment with a nitric acid solution 6 M for 6 h at its boiling temperature.

The physicochemical characteristics of activated carbons were determined previously, the results are presented in other work [[1], [2]] and are reported in Table 1.

### 2.2 Adsorption experiments

Phenol solutions were prepared with analytical reagent 99% purity (Merck, Germany) and distilled water, in concentrations ranging from 0.71 to 10.6 mmol L^−1^.

For the determination of phenol adsorption isotherms, 100 mg of each activated carbon was weighed in amber glass containers and 0.025 L of phenol solution was added. The containers were kept at constant temperature (298 K) under stirring until equilibrium was reached. Then, the solutions were filtered and the equilibrium concentration determined by UV–vis spectrophotometry on a GENESYS 10 UV–vis scanning apparatus (Thermo Fisher Scientific, Madison, WI, USA) at a 268 nm, maximum wavelength. The experimental data were fitted with the statistical program SigmaPlot 10® (Systat Software Inc., San Jose, CA, USA). The Equation (1) is used to determine the adsorbed quantity of phenol on activated carbon.

(1) $$Q=\frac{\left(C_{o}-C_{e}\right)V}{m}$$

where Q is the adsorbed quantity, Co represents the initial concentrations and Ce equilibrium concentrations of phenol. V represents the volume used in the containers and m the mass of activated carbons used in each experiment.

The experiments were repeated twice in each activated carbon.

### 2.3 Thermodynamic study

#### 2.3.1 Immersion calorimetry

Immersion enthalpies of activated carbon in phenol solutions were carried out in a Tyan type heat conduction microcalorimeter, which was equipped with a stainless steel cell of 0.015 L capacity, in which 0.010 L of the phenol solution was placed. A quantity of 0.10 g of each activated carbon was weighed in a glass ampoule with a fragile tip and placed in the calorimetric cell. The electric potential was recorded until baseline, the ampoule was broken and them the increase in potential due to wetting of the solid was recorded. The return of the potential at baseline was expected. Finally, an electrical calibration was made to determine the calorimeter constant.

The procedure was repeat used water to determine the immersion enthapies in the solvent.

Once the calorimetry was finished Equations (2), (3) are used to determine the immersion enthalpy.

(2) $$Q_{imm}=K_{cal}\left(watts\;V^{-1}\right)\text{∗ }area\;under\;the\;immersion\;cur\text{v}e$$

(3) $$\text{Δ}H_{imm}=\frac{Q_{imm}\left(J\right)}{Weight\;of\;acti\text{v}ated\;carbon\left(g\right)}$$

where K_cal_ represents the calorimeter constant, Q_imm_ is the immersion heat and ΔH_imm_ is the immersion enthalpy.

The experiments were repeated three times.

#### 2.3.2 Interaction enthalpy

The interaction enthalpy (ΔH_int_) corresponds to the enthalpy associated with activated carbon-phenol interaction, was determined from the application of the Hess law, assuming that the solutions are infinitely diluted. To avoid errors in the calculation, we used concentration higher than 0.74 mmol L^−1^, at concentrations below of this, the energy exchange is associated with the water-activated carbon interactions. Equation (4) shows the mathematical expression used to determine the enthalpy of interaction in this study.

(4) $$\text{Δ}Hint_{\text{AC}-\text{Phenol}}=\left(\text{Δ}Himm_{Phenol}\right)-\left(\text{Δ}Himm_{Water}\right)$$

#### 2.3.3 Gibbs energy and entropy changes

The Gibbs energy (ΔG) represents the energy available in the system to carry out the adsorption process. In turn, the Second Law of Thermodynamics also indicates the spontaneity and stability of the process when it is related to the thermodynamic equilibrium constant (K_a_). According to this, Equation (5) is used to calculate the Gibbs energy change [3].

(5) $$\text{Δ}G=-RTLnKa\;where\;Ka=\frac{C_{e}}{C_{o}}$$

The values of entropy changes (ΔS) were calculated from application of the Gibbs-Helmholtz equation using the immersion enthalpies and the Gibbs energy data. Equation (6) is the mathematical expression used to calculate de entropy. The experiments were made at 298 K.

(6) $$\text{Δ}S=\frac{\text{Δ}G-\text{Δ}H}{T}$$
